# Commissioning and evaluation of a new commercial small rodent x-ray irradiator

**DOI:** 10.2349/biij.2.1.e10

**Published:** 2006-01-01

**Authors:** MK Woo, RA Nordal

**Affiliations:** 1 Departments of Radiation Oncology and Medical Biophysics, Sunnybrook and Women's College Health Sciences Centre, University of Toronto, Canada; 2 UAB Comprehensive Cancer Center, Birmingham, Alabama, United States

**Keywords:** Animal irradiator, rodent irradiation, radiobiology, radiation beam measurement

## Abstract

An appropriate radiation source is essential in studies of tissue response in animal models. This paper reports on the evaluation and commissioning of a new irradiator suitable for studies using small animals or cell culture. The Faxitron is a 160-kVp x-ray machine that was adapted from an x-ray imaging unit through modifications to facilitate experimental irradiation. The x-ray unit is housed in a shielded cabinet, and is configured to allow multiple irradiation positions and a range of field sizes and dose rates. Use of this machine for animal irradiation requires characterisation of relevant dosimetry, and development of methodology for secondary beam collimation and animal immobilisation. In addition, due to the limitation of the irradiator, the optimal selection of three characteristics of the x-ray beam is important. These three characteristics, namely, the dose rate, the beam uniformity, and the field size are inter-dependent and the selection of a combination of these parameters is often a compromise and is dependent on the application. Two different types of experiments are selected to illustrate the applicability of the Faxitron. The Faxitron could be useful for experimental animal irradiation if the experimental design is carried out carefully to ensure that accurate and uniform radiation is delivered.

## INTRODUCTION

Animal models are of key importance in experimental radiation research, and accurate partial or whole body irradiation is critical for many types of investigations [1-4]. For example, delivery of specific doses to tumours is necessary in assessing tumour radiation response. Dose response curves for late radiation injury are often quite steep, and thus inattention to dosimetry can result in failure to reproduce expected effects [5-6]. Animal irradiation has been performed using appropriately collimated sealed source radioisotope machines. However, there is increasing interest in delivering organ-specific or whole body irradiation using x-ray units. There are several advantages of the latter. Firstly, facility-licensing requirements are much less stringent for x-ray machines. In addition, although clinical cobalt units have been used for animal irradiation, a dedicated radiation unit for experimental work is usually necessary because infection control practices within large animal facilities often preclude the transfer of animals between the animal colony and a remotely located irradiator. Other factors such as lower costs, smaller irradiator footprint, and easier maintenance, also add to the advantages of an x-ray unit.

The use of x-ray units does, however, present certain challenges. A major challenge is the more complicated dosimetry resulting from a lower beam energy and hence shallower penetration into tissue, leading to the possibility of non-uniformity of delivered dose [7]. While depth dose characteristics of x-ray beams are improved at longer treatment distances, the limited dose rate capabilities of these irradiators represent another challenge when appropriate treatment distances are employed. While radioisotope units have been available for some time, there is limited experience with commercial x-ray irradiators, and the question naturally arises as to whether x-ray irradiators can replace an isotope irradiator for animal irradiation experiments. This work reports on the design of irradiation conditions for partial and whole body animal irradiation using the Faxitron irradiator. This evaluation validates the suitability of the Faxitron for laboratory rodent irradiation experiments, and predicts its suitability for irradiation experiments using cell culture and other systems.

## MATERIALS AND METHODS

**Figure 1:**
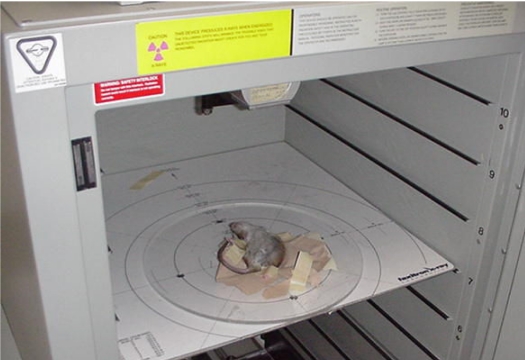
Interior of Faxitron cabinet. An anesthetised rat is placed on a tray mounted at shelf position 7. The x-ray tube can be seen at the top.

The Faxitron model CP160 (Faxitron X-Ray Corp., Wheeling, IL, USA) is a commercially available x-ray tube machine that is designed for animal irradiation. It was developed based on the modification of the company's existing line of imaging machines. The unit contains an x-ray tube mounted on the ceiling of a steel cabinet measuring 85 cm x 85 cm x 110 cm. The x-ray tube has a filter holder beyond the exit window for placement of additional filtration. For these experiments an added filtration of 0.8 mm Al was used. Inside the cabinet, eight tray guide positions accommodate a range of source-to-sample surface (SSD) distances varying from 13 to 99 cm, with a maximum field size coverage of 72 cm diameter. Additional guides can be affixed to the plexiglass tray for positioning of an animal jig or specimen. The unit can deliver x-rays at a maximum energy of 160 kVp and at a current of 6.3 mA. Power, current, and time settings are specified through a control panel on the front of the unit. Programming of a pre-stored set of kVp, mA, and time settings is possible. Timer settings are adjustable in increments of 0.1 minute. Localisation of the beam is achieved through alignment markings on the sample tray. Detailed specifications can be found at the website (http://www.faxitron.com). Figure 1 is a photograph of the unit with a rat placed on the tray.

Three characteristics of the radiation beam are important for the requirements of the irradiation experiments. The first is the dose rate of the radiation beam, which heavily affects the amount of time required for the experiment. The second is the field size of the beam, which could limit, perhaps, the number of animals that could be simultaneously irradiated. The third is the uniformity of the dose deposition, in the depth direction, i.e., in the direction that the beam travels through the tissue. In general, a uniform dose is desirable in the depth direction of the tissue, so that when a deep-seated tumour receives the required dose any normal tissue above it would not unnecessarily get a much larger dose.

It would be most desirable to have an x-ray beam that can deliver a uniform depth dose with a large field size and a high dose rate. Unfortunately, these parameters are inter-related so that usually increasing one parameter will compromise the others. For example, the dose rate could be increased by moving the specimen closer to the x-ray source (smaller source-to-surface distance (SSD)). However, this would reduce the maximum field size and also increase the dose non-uniformity in the depth direction. The selection of the appropriate irradiation parameters then depends heavily on the particular type of irradiation experiments, subject to the capability of the beam characteristics of the irradiator.

The Faxitron was then evaluated in the context of the above concept by applying the unit to two types of experiments in our laboratory. The first type is a small field irradiation of a single animal, and the second is the whole body irradiation of a group of animals, where the animals are either mice or rats. An example of the first type of experiments is the irradiation of a section of the spinal cord; for the purpose of studying radiation-induced response [2,4] and an example of the second type of experiments is the ablation of bone marrow [1,3]. For the small field irradiation experiments, the dose uniformity across the target volume in the depth direction of the beam is not a problem due to the small volume, but the depth of the target from the surface might result in a large dose delivered to the surface, especially if the SSD is small. Hence, a configuration with a parallel-opposing pair of beams, similar to that in standard radiation therapy, is used to obtain a uniform dose distribution in the depth dimension. For the whole-body irradiation experiments, the requirements are much more stringent. The field size has to be large enough to cover the whole animal, and preferably even larger to cover multiple animals. Moreover, the dose across the whole body in the depth direction has to be uniform to within 10%. In addition, the irradiation time need to be kept as short as possible to improve efficiency and to minimise the confinement of the animals. Again, the parallel-opposing beam configuration is used.

In the commissioning of the Faxitron, measurements of the beam characteristics for the two types of experiments were carried out in a phantom and these are reported below to demonstrate the capability of the Faxitron for these experiments. The depth-dose data for 3 different SSD's (9, 19 and 33 cm respectively) were measured using a Markus type parallel-plate ion-chamber (PTW-Freiburg, Freiburg, Germany) overlaid with sheets of tissue equivalent material (Solid WaterTM phantom material, Gammex RMI, Middleton, WI, USA). The absolute dose was determined using the AAPM TG-61 protocol using a cross-calibration procedure with the orthovoltage machine in the clinic. These depth-dose data were then used to calculate the dose uniformity across an animal with a thickness of 2 cm and 3 cm if a pair of parallel-opposing beams is used in an irradiation experiment.

In addition to the depth dose measurements, other physics measurements were carried out. These include measuring the beam uniformity in the cross-plane direction using film. Also, the stability of the dose rate with respect to delivery duration was assessed by running the beam continuously for an extended period of time (20 minutes). Lastly, the radiation level around the unit was surveyed using a Geiger counter.

In this paper only the measurements in phantom are reported. In the actual animal irradiation experiment, the pair of parallel-opposing beams would be delivered in two equal parts, with the second half to be delivered after flipping the animal or animal holder over. The animals used for the experiments are husbanded in the animal facility of the Sunnybrook and Women's College Health Science Centre, which is a laboratory animal colony accredited by the Canadian Council of Animal Care. All experimental protocols involving animals were approved by the Animal Care Committee of the Sunnybrook and Women's College Health Science Centre. The experiments will be reported in a separate paper, and the present work will only discuss the physics aspects on the commissioning of the Faxitron irradiation unit. An illustration of the Faxitron unit, as well as the process of the irradiation experiment, are included in the attached movie file (available for download with the online version).

## RESULTS

**Figure 2:**
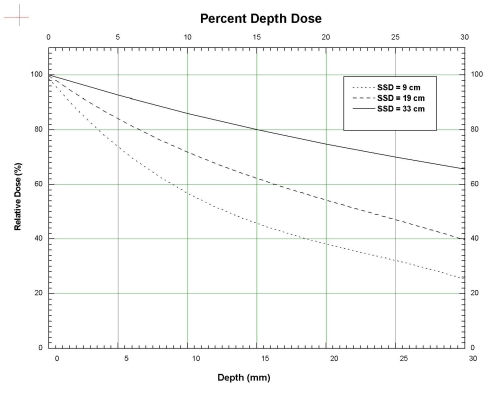
Percent Depth Dose curves for SSD = 9, 19 and 33 cm. The curves have been fitted to the measured data and smoothed. The uncertainties of the data are negligible. The curves have been normalised to 100% on the surface.

**Figure 3:**
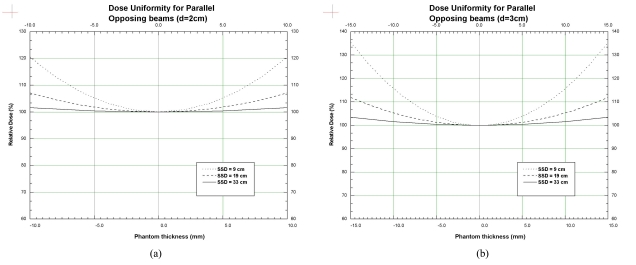
Dose variation in phantom with thickness, d of (a) 2 cm and (b) 3 cm, respectively. The curves have been normalised to 100% at the centre of the phantom.

The measured depth dose curves are shown in Figure 2, for the 3 SSD's of 9, 19 and 33 cm respectively. The data have negligible error bars (repeat measurements produce values that agree within less than 1%), and the curves have been fitted to the data and smoothed. Using these depth dose data, the calculated dose variation for a parallel-opposing pair of beams in the depth dimension for a phantom is shown in Figure 3a (for a phantom thickness of 2 cm) and Figure 3b (for a phantom thickness of 3 cm). For example, in Figure 3a, the dashed curve shows the dose variation in the depth direction for a phantom of thickness 2 cm, for an SSD of 19 cm, relative to 100% at the centre (0.0 mm) of the phantom. The dose on the surface (-10 mm and 10 mm) is around 107%. Similarly, the dotted line shows that the surface dose for an SSD of 9 cm is very high, up to 120% relative to the mid-point dose. The surface dose for an SSD of 33 cm is about 101.5%, showing good dose uniformity throughout the depth of the phantom. For the phantom thickness of 3 cm, in Figure 3b, the dose uniformity for the SSD of 19 cm is just over 10%, while that for an SSD of 9 cm is as high as 35%.

The trade-off in the dose uniformity for increased SSD is reflected in the decreased dose rate to the phantom. For an SSD of 19 cm in the 3 cm phantom, the dose rate was determined to be around 350 cGy/min. The corresponding dose rate for an SSD of 9 cm was 1500 cGy/min and that for the SSD of 33 cm was 160 cGy/min.

For other physics measurements, firstly, the beam uniformity in the cross-plane direction as determined using film shows that the useful area of the beam was substantially less than as indicated by the beam outline on the tray. For example, at the SSD of 33 cm, the indicated beam diameter on the tray was 26 cm, whereas the useful part of the beam where the uniformity was within 10% was confined to a diameter of 16 cm. Secondly, the stability of the dose rate over time was determined to be within 5% over a period of 20 minutes. And lastly, no detectable radiation level above background was measured outside the cabinet.

## DISCUSSION AND CONCLUSIONS

The above results show that the Faxitron could be configured to provide acceptable dose uniformity across animals of typical thicknesses of 2 or 3 cm, for both the small field and whole-body irradiation experiments. For the small field experiments such as investigation of spinal cord radiosensitivity, a typical dose prescription is around 18 Gy, resulting in an irradiation time of about 3 minutes per side (SSD=19cm). For the whole-body experiment, a typical dose for bone-marrow ablation is 8 Gy, resulting in an irradiation time of 2.5 minutes per side (SSD=33cm). Only one single rat could be covered in the whole-body irradiation at a time, but for smaller mice about 3 animals could be irradiated at the same time.

The fact that the animal has to be turned over means that the animal has to be immobilised or anesthetised. A significant improvement then would be to install a second x-ray tube on the bottom of the cabinet to provide a parallel-opposed pair, which would additionally halve the required time.

The low energy of the beam and the resulting steep dose fall-off need to be carefully considered when assessing the applicability of the Faxitron for any irradiation experiment. One example is the irradiation of cells in suspension, since the specimen cannot be turned over to achieve a uniform dose distribution. A solution in a flask is also a problem.

Basically, the Faxitron can be considered to be an ortho-voltage x-ray unit and the dosimetry of it conforms to the AAPM protocol for beams of this energy range [8]. With careful experimental design, it can provide a solution as a cost-effective irradiator with minimal radiation-protection concerns.
